# Prediction of a typhoon track using a generative adversarial network and satellite images

**DOI:** 10.1038/s41598-019-42339-y

**Published:** 2019-04-15

**Authors:** Mario Rüttgers, Sangseung Lee, Soohwan Jeon, Donghyun You

**Affiliations:** Pohang University of Science and Technology, Department of Mechanical Engineering, Pohang, 37673 Korea

## Abstract

Tracks of typhoons are predicted using a generative adversarial network (GAN) with satellite images as inputs. Time series of satellite images of typhoons which occurred in the Korea Peninsula in the past are used to train the neural network. The trained GAN is employed to produce a 6-hour-advance track of a typhoon for which the GAN was not trained. The predicted track image of a typhoon favorably identifies the future location of the typhoon center as well as the deformed cloud structures. Errors between predicted and real typhoon centers are measured quantitatively in kilometers. An averaged error of 95.6 km is achieved for tested 10 typhoons. Predicting sudden changes of the track in westward or northward directions is identified as a challenging task, while the prediction is significantly improved, when velocity fields are employed along with satellite images.

## Introduction

Every year tropical cyclones cause death and damage in many places around the world. Cyclones are formed when water at the sea surface becomes warm, evaporates, and rises in a form of clouds, and while it cools down, the condensation releases strong energy in a form of winds. Rotation of the earth gives a cyclone its spinning motion. At the center usually a hole forms, which is called an eye of the cyclone. At the eye, the pressure is low and energetic clouds and winds get attracted. Warm air can rise through the hole and, favored by high altitude winds that can create a suction effect, increase the energy of the system. Crosswinds, vertical wind shear or dry air, on the other hand, can block this mechanism^1^. Depending on water conditions and surrounding winds tropical cyclones and their associated storm surges can be a great danger when they find their way to a populated land.

The destructive force of a tropical cyclone comes from the wind speed of the rotating air and the rainfall that can cause disastrous flooding^2^. The highest wind speeds are usually found near the typhoon center. Strong rain, on the other hand, can be experienced near dense cloud structures. Therefore, the track of a cyclone is characterized by both the coordinate of the typhoon center and the shape or the distribution of clouds. Sun *et al*.^3^ found that tropical cyclones are becoming stronger, larger, and more destructive in the context of global warming. Kossin^4^ mentions in his study that translation speeds of tropical-cyclones have decreased globally by 10% and in Asia by 30% over the period 1949–2016. The lower the velocity of the cyclone as a whole system, the more rain falls on one location and the higher the risk for flooding. Thus, slower translation speeds can cause even more destructive floods.

The present work concentrates on tracks of cyclones that form in the north-western Pacific Ocean, also known as typhoons. The geographical focus lies on the Korean peninsula. In the past, Korea has suffered from numerous typhoons of different sizes. Typhoon Sarah was the strongest one. The cyclone hit the island Jeju on September 16th in 1959 during the traditional Thanksgiving festival leaving 655 dead, 259 missing, and more than 750,000 homeless^5^. In the younger history of the country, especially typhoons Rusa (August 2002) and Maemi (September 2003) have caused fear and death among the Korean population. Together they have been responsible for 376 casualties and a damage of 11.5 billion USD^6,7^. Xu *et al*.^8^ investigated paths of super typhoons approaching China over the past 50 years. They identified a trend of strong typhoons getting attracted by the moist environment in southeastern China. This trend also affects damages on the Korean peninsula, since the size of a super typhoon can be similar to the spatial distance between the Chinese east coast and the Korean west coast. To save lives and reduce such damages in the future, accurate forecast methods need to be established.

Accuracy is only one criterion when talking about the prediction of natural disasters. Speed and flexibility play a significant role as well. Forecasts should be done quickly and forecast tools should be able to react immediately on sudden changes. Finally they should be inexpensive. Current predictions in South Korea are done by conducting numerical simulations on a Cray XC40 supercomputer with 139,329 CPUs. This method consumes a huge amount of computational time. The maintenance costs for such expensive hardware are also very high. An efficient forecast method that considers all these criteria is necessary.

In the work of Kovordanyi and Roy^9^ nine existing forecast techniques are listed. Subjective assessment is an evaluation of the cyclone’s behavior based on large-scale changes of the flow field. In analogue forecasts, features of the cyclone are compared to all previous storms in the same region and the movement is derived from past experiences. In the third technique, the steering current is estimated by analyzing winds at certain locations and altitudes. The statistical technique uses regression analyses, whereas the dynamical technique uses numerical modeling and simulation. Besides computer based approaches, empirical forecasting based on experiences of meteorologists are considered as a good complement to other techniques. The persistence method allows short-term predictions, but relies on the cyclone to keep its recent track. Satellite-based techniques use satellite images to make forecasts of the track and intensity of tropical cyclones based on cloud patterns. Finally, combinations of the previously mentioned approaches are defined as hybrid techniques.

In this study a satellite-based technique is combined with a deep learning method. Lee and Liu^10^ probably firstly used satellite images of tropical cyclones for track prediction with the help of neural networks. But images did not function as input data for the network. In a pre-processing step, information like the Dvorak number, the maximum wind speed or the cyclone’s position were extracted from images and fed to the network for a time-series prediction. The model has shown improvements of 30% over the bureau numerical tropical cyclone prediction model used in Guam for forecasting tropical cyclone patterns and tracks. Kovordanyi and Roy^9^ were the first who actually used satellite images as input data for a neural network. The network favorably detected the shape of a cyclone and predicted the future movement direction. Hong *et al*.^11^ utilized multi-layer neural networks to predict a position of the cyclone’s eye in a single high-resolution 3D remote sensing image. The network learns coordinates of an eye from labeled images of the past data and predicts them in test images. Kordmahalleh *et al*.^12^ have used a sparse recurrent neural network (RNN) to predict trajectories of cyclones coming from the Atlantic Ocean or the Caribbean Sea, also known as hurricanes. They used dynamic time warping (DTW), which compares the trajectory of a target hurricane to all hurricanes of a dataset. For the prediction process, only hurricanes which have similarities in trajectories to that of the target hurricane, were used. One drawback is that they assumed monotonic behaviour, which means that a hurricane never comes back to a location where it was before. In reality this is not always the case. Alemany *et al*.^13^ also used an RNN to predict hurricane trajectories, but instead of assuming monotonic behaviour they considered all types of hurricanes. They used a grid-based RNN that takes the wind speed, latitudinal and longitudinal coordinates, the angle of travel, and the grid identification number from past motions of the hurricane as inputs. With the grid identification number, spatial relations on a map were learned. Their forecasts could achieve a better accuracy than results of Kordmahalleh *et al*.^12^. Zhang *et al*.^14^ utilized matrix neural networks (MNNs) for predicting trajectories of cyclones that occurred in the South Indian ocean. They stated that MNNs suit better to the task of cyclone trajectory prediction than RNNs, because MNNs can preserve spatial information of cyclone tracks.

In the previous research, deep learning methods were used to identify the center of a cyclone in a satellite image or to predict a trajectory of a cyclone using discrete meteorological data without cloud images. None of the previous studies using neural-networks reported the capability of predicting both the coordinate of a typhoon center and future shape of cloud structures around the typhoon. But, as mentioned before, a safe warning system should be able to forecast both, cyclone centers and their area of impact in a form of cloud structures. To the best of authors’ knowledge, a combination of satellite images and a deep learning method has not yet been used to predict both future typhoon centers and the future cloud appearance. In this study, a generative adversarial network (GAN) is applied for both tasks. As an input it uses chronologically ordered satellite images from the past and as an output it creates images that show the typhoon hours ahead.

A conceivable alternative to the present approach of predicting the coordinate of a typhoon center can be predicting the direction and the speed of a typhoon, from which, afterwards, the future position of the typhoon center is calculated. However, to predict the moving direction and the speed of a typhoon using a GAN, it is still necessary first to forecast the typhoon center, where the direction and the speed of the typhoon are defined. The present GAN employs satellite cloud images along with a marked typhoon center as the input set, but without explicit information about the moving direction or the speeds at the typhoon center.

Predictions can be generated within seconds using a single graphics processing unit (GPU). Thus, the method is significantly quicker and cheaper than the conventional methods, like numerical simulations, where hundreds of thousands CPU are used for one simulation. Tracks of typhoons can be predicted on a real time basis by updating input data as a function of real time. While relearning an updated whole dataset with new inputs is not necessary, training the network with the updated dataset can be conducted on a real time basis by employing a fine-tuning technique or by using multiple GPUs. The GAN is a deep learning technique used to generate samples in forms of images, music or speech. It has been introduced by Goodfellow *et al*.^15^, who successfully generated new samples to the MNIST and CIFAR-10 image datasets. Their multi-scale convolution network architecture has originally been proposed by Mathieu *et al*.^16^ for image prediction of a video sequence. The inspiration for the present study comes from a problem in fluid dynamics. Lee and You^17^ used a GAN to successfully predict unsteady flow over a cylinder. They reported that the adversarial training of the GAN leads the network to generate better prediction of flow fields by extracting flow features in an unsupervised manner, compared to convolutional neural networks.

## Results

The GAN has been trained and tested on an NVIDIA Tesla K40c GPU. Ten typhoons in the test dataset are: Faye (Juli 1995), Violet (September 1996), Oliwa (September 1997), Saomai (September 2000), Rammasun (June/July 2002), Maemi (September 2003), Usagi (July/August 2007), Muifa (July/August 2011), Neoguri (June/July 2014), and Malakas (September 2016). The testing for each typhoon is done in sequences. One sequence contains chronologically ordered input images, the ground truth image, and the generated image. A sequence is named with the date and time (universal time coordinated - UTC) of the corresponding ground truth image, for example: “1993072718” stands for 27th of July in 1993 at 6 pm (UTC).

### Prediction of the typhoon center

**Figure 1:**
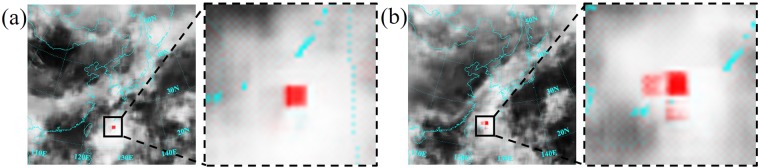
(**a**) Clearly identifiable predicted typhoon center. (**b**) Three possible predicted typhoon centers.

The accuracy in the prediction of the typhoon center coordinate is measured by comparing the labeled red square of the ground truth image with the predicted red square of the generated image, rather than coordinates in the Cartesian space. This is because convolutional neural networks have difficulty in learning a mapping between the coordinate in the Cartesian space and the coordinate in the one-hot pixel image space^18^. The red square in the predicted image is detected by applying a color filter on the generated image and a convolution with the size of the red square on the filtered image. The pixel with the highest value in the convolved image gives the location of the predicted typhoon center. In most of the cases images with a clearly identifiable red square are generated, as shown in Fig. 1(a). In some cases, however, the GAN provides several alternatives, as visualized in Fig. 1(b). Each possible predicted typhoon center has its own color strength. The color intensities can be interpreted as probabilities; a strong red square shows a confident prediction, a weak mark stands for a possible predicted typhoon center with a low probability. In cases like in Fig. 1(b), the location with the strongest color intensity is chosen. After identifying the (*x*, *y*)-pixel coordinates of the predicted typhoon center, they are transformed back to latitudinal (*ϕ*) and longitudinal (*λ*) coordinates via georeferencing.

**Figure 2:**
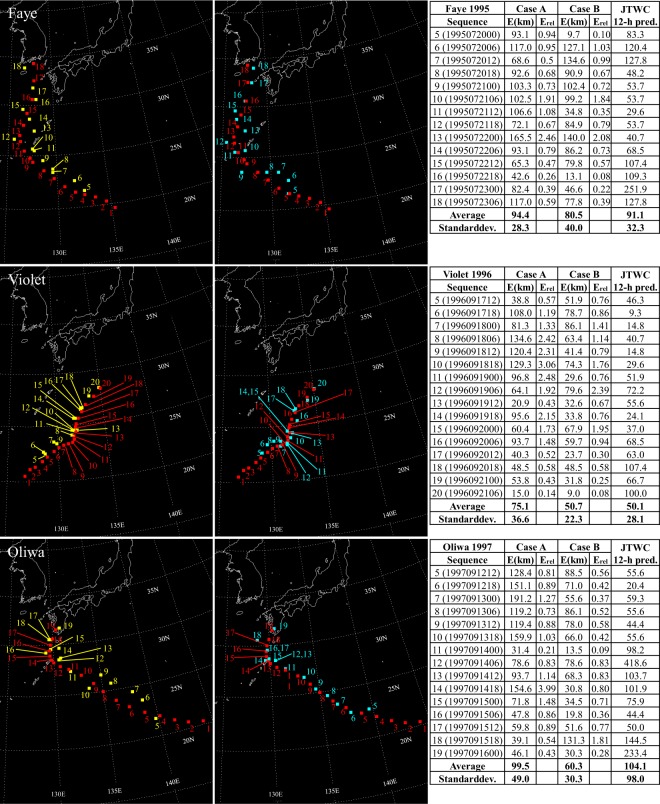
For typhoons Faye, Violet, and Oliwa, predictions using satellite images only (Case A) are shown in the left column with yellow squares, and predictions using satellite images along with velocity fields at 10 m height (Case B) are shown in the right column with blue squares. For both cases, center coordinates of the real typhoon are represented by red squares. Absolute errors, relative errors, and errors in kilometers for 12-hour predictions reported in annual tropical cyclone reports of the Joint Typhoon Warning Center (JTWC)^19–21^ as a function of the sequence are summarized in tables.

**Figure 3:**
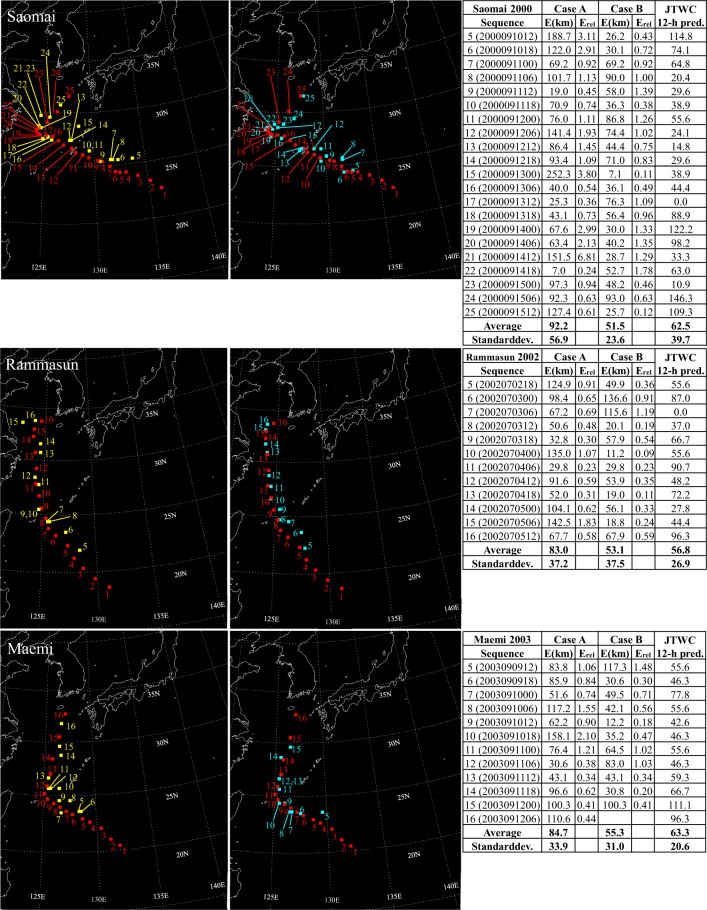
For typhoons Saomai, Rammasun, and Maemi, predictions using satellite images only (Case A) are shown in the left column with yellow squares, and predictions using satellite images along with velocity fields at 10 m height (Case B) are shown in the right column with blue squares. For both cases, center coordinates of the real typhoon are represented by red squares. Absolute errors, relative errors, and errors in kilometers for 12-hour predictions reported in annual tropical cyclone reports of the Joint Typhoon Warning Center (JTWC)^22–24^ as a function of the sequence are summarized in tables.

**Figure 4:**
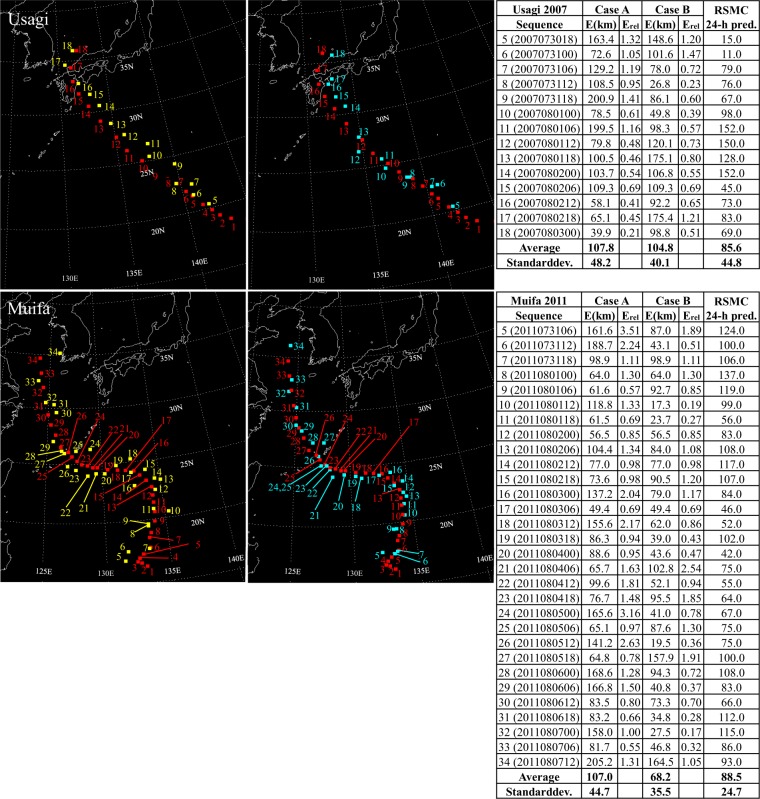
For typhoons Usagi, and Muifa, predictions using satellite images only (Case A) are shown in the left column with yellow squares, and predictions using satellite images along with velocity fields at 10 m height (Case B) are shown in the right column with blue squares. For both cases, center coordinates of the real typhoon are represented by red squares. Absolute errors, relative errors, and errors in kilometers for 24-hour predictions reported in annual reports on the activities of the Regional Specialized Meteorological Center (RSMC) Tokyo - Typhoon Center^25,26^ as a function of the sequence are summarized in tables.

**Figure 5:**
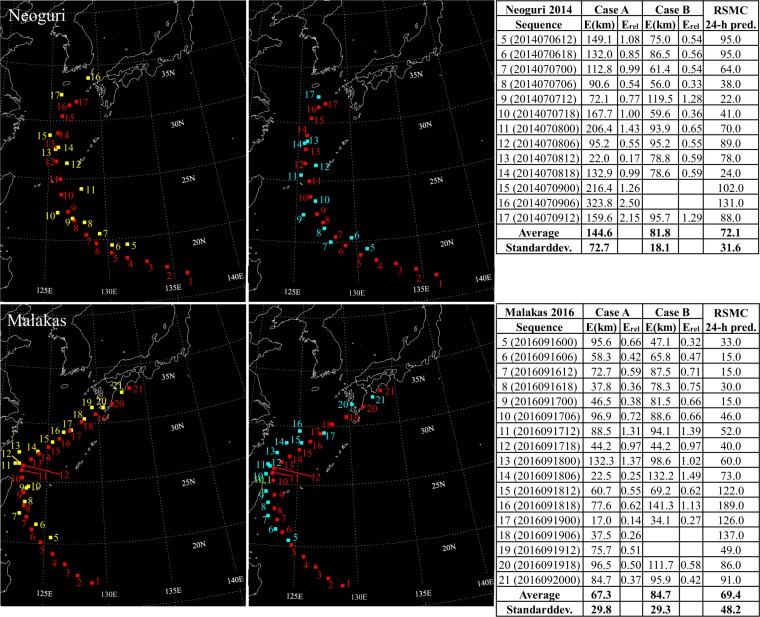
For typhoons Neoguri, and Malakas, predictions using satellite images only (Case A) are shown in the left column with yellow squares, and predictions using satellite images along with velocity fields at 10 m height (Case B) are shown in the right column with blue squares. For both cases, center coordinates of the real typhoon are represented by red squares. Absolute errors, relative errors, and errors in kilometers for 24-hour predictions taken from annual reports on the activities of the Regional Specialized Meteorological Center (RSMC) Tokyo - Typhoon Center^27,28^ as a function of the sequence are summarized in tables.

Results for ten test typhoons are presented in Figs 2–5. Images in the left column of the figures are predicted by using only satellite images as input data, and are referred to Case A, where locations of the typhoon center are marked with yellow squares. Images in the right column of the figures are predicted by using satellite images along with velocity fields at 10 m height as input data, and are referred to Case B, where locations of the typhoon center are marked with blue squares. Results in both cases are contrasted to locations of the real typhoon center, represented by red squares. Tables in the figures contain absolute errors, relative errors, and errors of forecasts that have been conducted by the Joint Typhoon Warning Center (JTWC) for typhoons Faye, Violet, Oliwa, Saomai, Rammasun, and Maemi^19–24^, and by the Regional Specialized Meteorological Center (RSMC) Tokyo - Typhoon Center for typhoons Usagi, Muifa, Neoguri, and Malakas^25–28^.

Focusing firstly on Case A, it is noticeable how errors increase, when typhoons experience sudden northward or westward course changes. It is observed for all ten test typhoons. Figure 2 shows increased errors when typhoon Faye turns northward at sequences 10, 11, and 13, and similarly for Violet at sequences 8–11 and Oliwa at sequences 13–15. In Fig. 3, similar observations are made. When typhoon Saomai changes its path westward at the sequence 15 and northward around the sequence 21 errors increase noticeably. Typhoons Rammasun and Maemi also show difficulty in the prediction when they get deflected northward at sequences 10 and 8–10, respectively. Figures 4 and 5 show results for typhoons Usagi, Muifa, Neoguri, and Malakas. The dragon shaped typhoon Muifa underwent sudden course changes at sequences 16–18 or 24–26. The youngest typhoon among the ten test cases, Malakas, shows the highest error at the sequence 13, when its course has a sudden change to the north.

**Figure 6:**
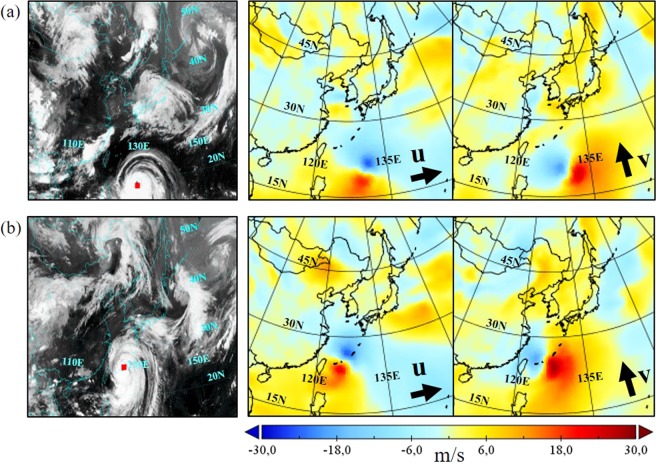
Satellite images and velocity fields at 10 m height of typhoon Neoguri at sequences 5 (**a**) and 11 (**b**). Black arrows indicate directions of positive zonal and meridional velocity components. (Source of satellite images: Korean Meteorological Administration (KMA). http://www.weather.go.kr/weather/images).

The path of a typhoon is highly influenced by steering flow that is controlled by storms’ ambient environment. Sometimes, when a storm is surrounded by multiple systems whose circulations compete to each other, the steering flow is difficult to predict. As a results, the storms’ track is hard to predict. Wu *et al*.^29^ mention that typhoons in the Northwestern Pacific usually experience sudden northward or westward course changes. In the north-turning case, winds are enhanced on the southeast side of tropical cyclones, in west-turning cases, north-easterly winds are enhanced on the west side. One example is illustrated in Fig. 6, which shows satellite images and velocity fields for typhoon Neoguri at sequences 5 (Fig. 6(a)) and 11 (Fig. 6(b)). At the sequence 11, when the typhoon experiences a northward deflection, strong winds are noticed at the southeast side of the center, compared to the velocity field at the sequence 5. It may be difficult for the GAN to learn such phenomena only from satellite images.

Results in Case B indicate that combining satellite images with images of surface velocity fields has a positive impact on typhoon predictions. Although surface winds cannot represent the complete steering flow, they contain enough information to reduce the average error for all test typhoons, except for typhoon Malakas. Especially, the previously mentioned northward or westward course changes are predicted much more accurately in Case B. Except for results for typhoons Faye and Rammasun, standard deviations of the error are also reduced for all test typhoons. Therefore, adding information of the velocity field to the network seems to be beneficial to improving the predictive capability of the present GAN-based method.

Several findings are gained by analyzing errors in predictions conducted by the JTWC and the RSMC. Firstly, it is observed that for typhoons Faye, Oliwa, Saomai, Rammasun, Maemi, and Muifa, errors in Case B are lower than errors of 12-hour predictions conducted by the JTWC and 24-hour forecasts of the RSMC. Secondly, six of the ten test typhoons show lower standard deviations for results in Case B compared to predictions reported by the JTWC and the RSMC. This may be partly due to the shorter prediction interval (6-hours) in the present study than those of the JTWC and the RSMC. Thirdly, whereas sudden northward or westward deflections are the challenging regime in Case A, forecasts conducted by the JTWC and RSMC show no increased errors when sudden course changes occur. However, their challenging regime seems to be interactions between storms and land, since for all ten typhoons errors increase before landfall. Difficulties in predictions before landfall are noticeable in Case A for typhoons Faye, Saomai, Rammasun, Maemi, Muifa, and especially Neoguri. In Case B, these difficulties are handled much better by the GAN.

Table 1 compares the frequency distribution of *E* of Cases A and B for all sequences. For Case A, in 74.5% of the cases the error is less than or equal to 120 km, compared to 92.5% in Case B. In Case A, a combined 42.4% have an accuracy of less than or equal to 80 km, which could be increased to 64.8% in Case B. In Case B, only 12 sequences are found above 120 km. The total error in Case A, 95.6 km, could be reduced by 27.7% to 69.1 km in Case B.

**Table 1:** Frequency distribution for the prediction of all sequences for Case A (Only satellite images) and Case B (Satellite images combined with data of the velocity field at 10 m height).

Error (km)	Case A	Percentage (%)	Case B	Percentage (%)
0–40	18	10.9	41	25.9
41–80	52	31.5	62	38.9
81–120	53	32.1	44	27.7
≥121	42	25.5	12	7.5

### Prediction of the cloud shape

**Figure 7:**
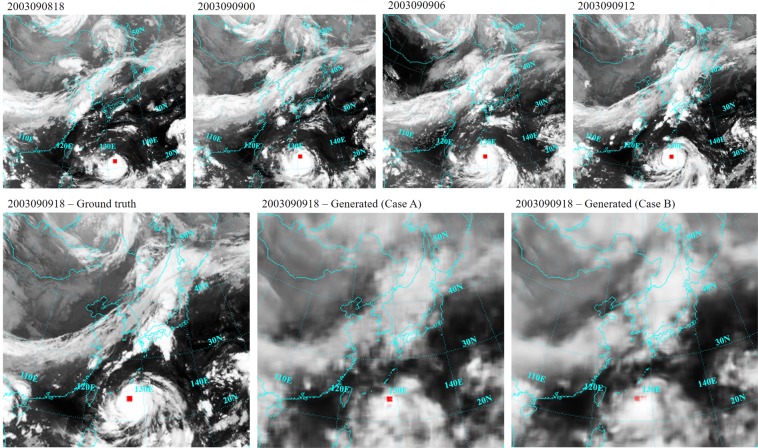
Prediction of the sequence 6 (2003090918) for typhoon Maemi (Source of satellite images: Korean Meteorological Administration (KMA). http://www.weather.go.kr/weather/images).

As an example for the generation of images that show the cloud shape, Fig. 7 illustrates the prediction for the sequence 6 of typhoon Maemi. Generated images do not have the same sharpness than the ground truth image, instead they are blurry. Blurriness is a well known challenge in video prediction tasks, which is tried to be overcome by using a gradient difference loss function in the present study. However, although generated images suffer from a certain degree of blurriness, the main structure of clouds is still visible. Furthermore, in the generated image in Case B the spinning motion of the typhoon seems to be reproduced more realistically than in the generated image in Case A.

**Figure 8:**
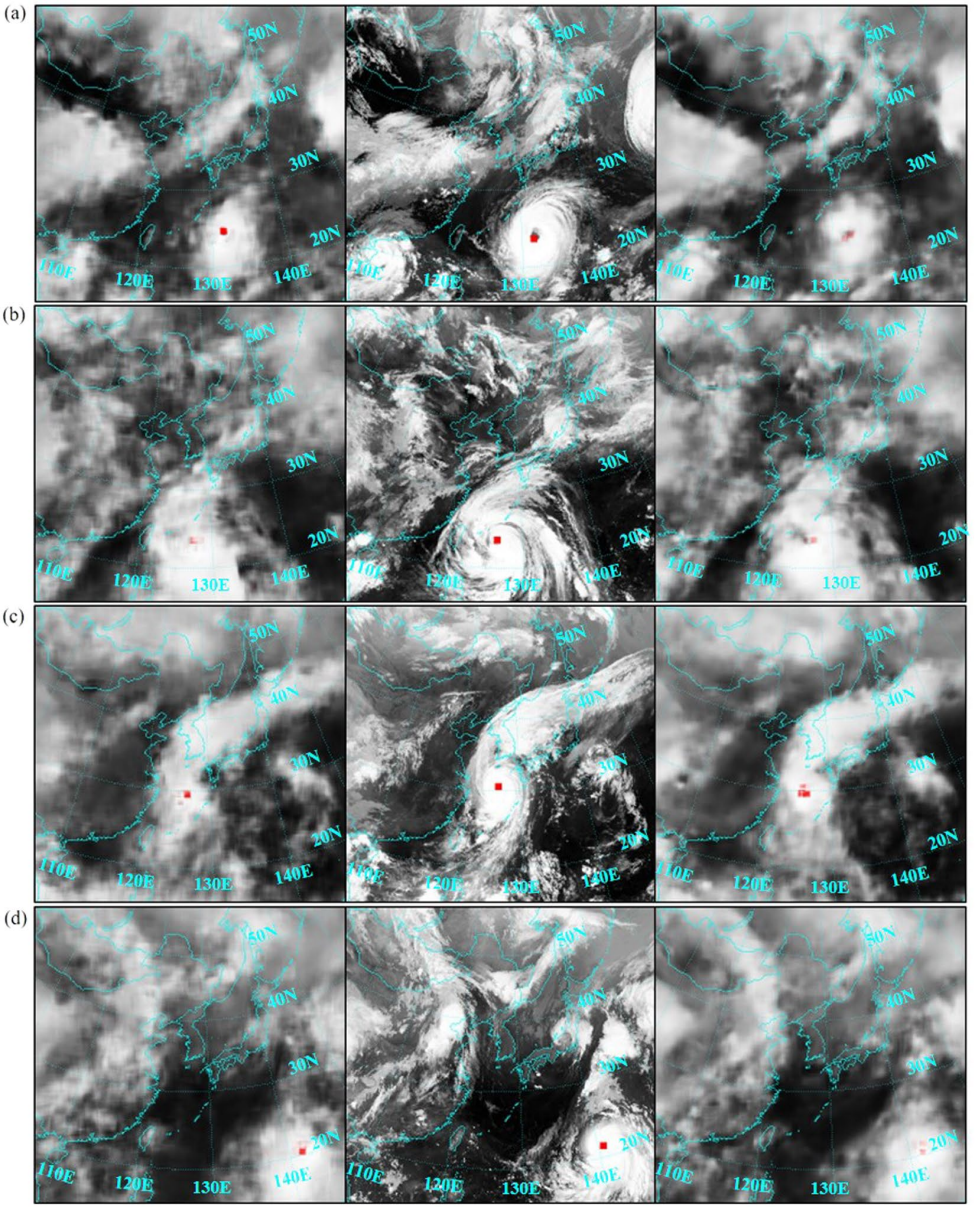
Generated images by using satellite images as input (Case A) in the left-most column, and images by using satellite images along with velocity fields at 10 m height (Case B) in the right-most column, compared to corresponding ground truth images in the middle column: (**a**) Sequence 11 of typhoon Violet, (**b**) sequence 6 of typhoon Rammasun, (**c**) sequence 15 of typhoon Maemi and (**d**) sequence 5 of typhoon Usagi (Source of satellite images: Korean Meteorological Administration (KMA). http://www.weather.go.kr/weather/images).

This is shown clearer in Fig. 8, where generated cloud images in Cases A and B of four randomly chosen sequences are presented. Generated images in Case A seem to be static and do not represent any dynamics. In the sequence 11 of typhoon Violet, for example, the image in Case A cannot reproduce the spinning motion of the typhoon (see Fig. 8(a)). The image in Case B, on the contrary, does not only illustrate the typhoon more realistically, but also cloud patterns in the remaining parts of the image. In the sequence 6 of typhoon Rammasun, the prediction in Case B again resolves the spinning motion of the typhoon much better than the prediction in Case A (see Fig. 8(b)). Details, like the cloud structure east or north of the typhoon center, are generated much more reliably. In the sequence 15 of typhoon Maemi the cyclone has almost reached the Korean peninsula. Parts of the cloud structure start to dissipate, but a significant part follows a strong westerly wind at a high altitude, called jet stream. In Fig. 8(c) it can be noticed how the result in Case B reproduces the suction effect of the jet stream in a smoother way than the predicted image in Case A. Finally, in Fig. 8(d) the image in Case B highlights much better how surrounding clouds move into the eyewall than the image in Case A.

## Discussion

The application of a deep learning method for typhoon track prediction in forms of typhoon center coordinates and cloud structures has been explored. Learning only from satellite images, 42.4% of all typhoon center predictions have absolute errors of less than 80 km, 32.1% lie within a range of 80–120 km and the remaining 25.5% have an accuracy above 120 km. The averaged error lies at 95.6 km. In general, errors increase when typhoons undergo sudden northward or westward course changes. Predictions in this challenging regime get much more accurate, when satellite images are combined with data of the velocity field at 10 m height. In that case, 64.8% of all predictions are below 80 km, 27.7% lie within a range of 80–120 km and only 7.5% remain with an accuracy above 120 km. The averaged error could be reduced by 27.7% to 69.1 km. Furthermore, it has been shown that the GAN is able to generate images that reproduce cloud appearance.

From a user’s perspective, it is helpful to make predictions in a quicker and cheaper manner. Current predictions in many countries rely on highly expensive numerical simulations using the state-of-the-art supercomputers, of which acquisition is limited by few advanced countries. Furthermore, it is expected that the present deep-learning-based method can be utilized in combination with other conventional techniques, especially, for non-user-biased analysis and decision making processes. Although, in the present study, predictions are conducted for a 6-hour interval, extension of the prediction time-interval is straightforward once the acquisition of more satellite images is possible. To improve the prediction accuracy, the next step will be adding physical information to the input data, like the sea surface temperature, the surface pressure, and velocity fields at various heights. Just learning from satellite images that show the cloud structure and the typhoon center is a good starting point but not sufficient for learning whole complex phenomena that are responsible for the creation and motion of typhoons.

## Methods

### Satellite images

**Figure 9:**
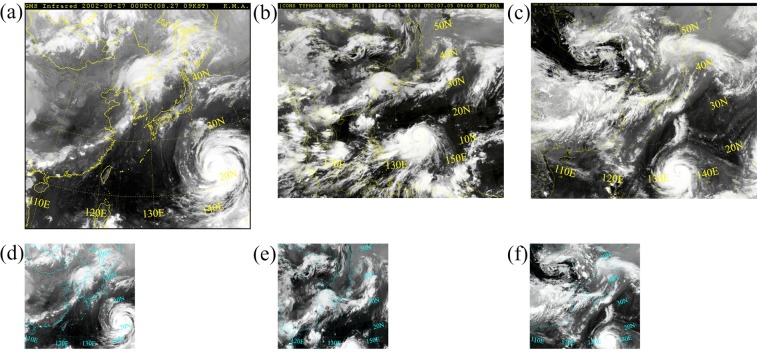
Images of typhoon Rusa in August 2002 as an example for the shots taken from 1993 to 2010 before (**a**) and after (**d**) preprocessing, images of typhoon Neoguri in July 2014 as an example for the shots taken from 2011 to 2014 before (**b**) and after (**e**) pre-processing and images of typhoon Goni in August 2015 as an example for the shots taken from 2015 to 2017 before (**c**) and after (**f**) pre-processing (Source of satellite images: Korean Meteorological Administration (KMA). http://www.weather.go.kr/weather/images).

Input satellite images, which were captured by satellites at the altitude of 35,786 km, have been provided by the Korean Meteorological Administration (KMA)^30^. They contain 76 typhoons from 1993 till 2017 that hit or were about to hit the Korean peninsula. Whereas Hong *et al*.^11^ used satellite images directly, in this study pre-processing of the images is inevitable. During the 25 years of capture time, different satellites have been operating. Thus, raw images have different perspectives on the Korean peninsula. Three different types of image perspectives and pixel sizes are provided (see Fig. 9). The network takes images with the same pixel size and learns better if all images have the same or a similar perspective on the north-western Pacific Ocean.

Figure 9(a) shows an image of typhoon Rusa in August 2002 as an example for the first perspective of the first period from 1993 till 2010. The images have a pixel size of 512 × 512. Initially, the images have been generated by the Geostationary Meteorological Satellite (GMS) number 4 of Japan^31^. The GMS-4 satellite was replaced by the GMS-5 satellite in 1996 with a design life of five years. But the replacement failed and the GMS-5 had to operate for three more years, before the US National Oceanic & Atmospheric Administration (NOAA) helped out with its GOES-9 Satellite in 2003. Two years later Japan could use the Multi-functional Transport Satellite (MTSAT) type 1 R until 2010. All satellites used the Visible and Infrared Spin-Scan Radiometer (VISSR) technique to obtain visible and infrared spectrum mappings of the earth and its cloud cover. The upper black bar, containing the satellite name, the date and the time written in yellow, as well as the black frame are unnecessary information for the learning phase. Furthermore, the pixel size causes memory issues in the test phase. Thus, images have been cropped and resized to 250 × 238 pixels (see Fig. 9(d)).

The perspective of images captured between 2011 and 2014 is presented in Fig. 9(b). The image with the pixel size of 512 × 412 shows typhoon Neoguri that formed in July 2014. It has been captured by Korea’s first multi-purpose geostationary meteorological satellite, namely Communication, Ocean and Meteorological Satellite (COMS). As in the previous case, the upper black bar is cropped and the images are resized to 250 × 238 pixels (see Fig. 9(e)). The view on the earth in Fig. 9(e) is similar to the view in Fig. 9(d).

An example for raw images of the remaining data is shown in Fig. 9(c). The operating satellite is the same like in the previous case. The 512 × 433 pixel shot shows typhoon Goni from August 2015. Except for a different thickness of the upper black bar, the pre-processing steps match with the previous case and lead to images like in Fig. 9(f).

All images have the ‘png’ format with three color channels (R(red)G(green)B(blue)). To improve the visibility of the country boarders, their color has been changed from yellow to blue. In total 1,628 images are stored, with a time step size of 6 hours between images. There are two accuracy criteria for the typhoon track prediction, a quantitative and a qualitative criteria. In the quantitative criterion, the difference between the coordinate of the predicted typhoon center and that of the ground truth is taken into consideration. In the qualitative criterion, the predicted shape of clouds and the shape of the ground truth cloud are compared.

**Figure 10:**
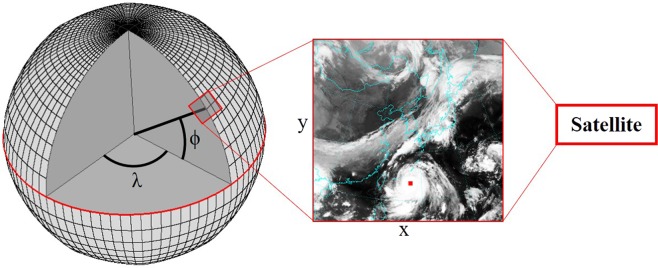
Georeferencing of the typhoon center coordinates (Source of the satellite image: Korean Meteorological Administration (KMA). http://www.weather.go.kr/weather/images).

Every satellite image has been labeled with a red square at the typhoon center. The latitudinal (*ϕ*) and the longitudinal (*λ*) coordinates of the typhoon centers are provided by the Japan Meteorological Agency (JMA)^32^. In order to label each satellite image with its red square, *ϕ* and *λ* coordinates have to be transfered to (*x*, *y*)-pixel coordinates in the image. This process is known as georeferencing and is illustrated in Fig. 10. Images are split into training and test data. The training data contain 1,389 images of 66 typhoons, the test data are 239 images of 10 typhoons.

### Velocity fields

**Figure 11:**
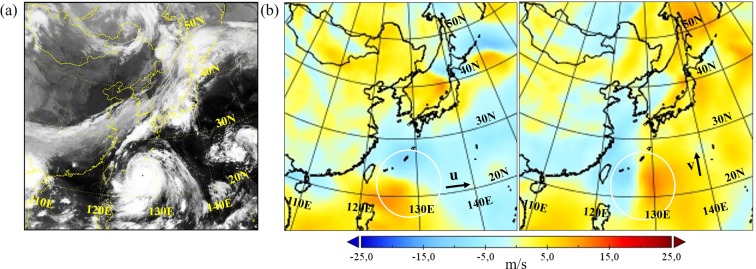
(**a**) Satellite image and (**b**) zonal [u] and meridional [v] velocity fields at 10 m height of typhoon Maemi on September 10th in 2003 at 0:00 Coordinated Universal Time (UTC) (Source of the satellite image: Korean Meteorological Administration (KMA). http://www.weather.go.kr/weather/images).

In order to help the GAN to better learn the movement of typhoons, the public dataset ERA-interim^33^ is employed. ERA-interim is a global atmospheric reanalysis starting from 1979 that has continuously been updated until today. It uses a fixed version of a numerical weather prediction system (IFS - Cy31r2) to produce reanalyzed data. The available data have time steps of 6 hours and their dates fit to the satellite images described in the previous section. Although ERA-interim data cover the whole globe, for this work only the area around the Korean peninsula is selected. Raw data with grid resolution of 0.75 degrees are refined to 0.125 degrees by applying linear interpolation, which leads to a resolution of nearly 13.8 km^34^. Images from reanalysis data are generated with the software Panoply and the map type Lambert Conformal Conic to match the view of the satellite images^35^. In the present study, reanalysis data are used by the GAN to learn information about the surface velocity field at 10 m height. Figure 11 gives an example for typhoon Maemi. The velocity field in Fig. 11(b) is contrasted with the satellite image in Fig. 11(a). Color changes in the zonal and meridional velocity components located in the white circles indicate a spinning motion of the typhoon.

### Deep learning methodology

**Figure 12:**
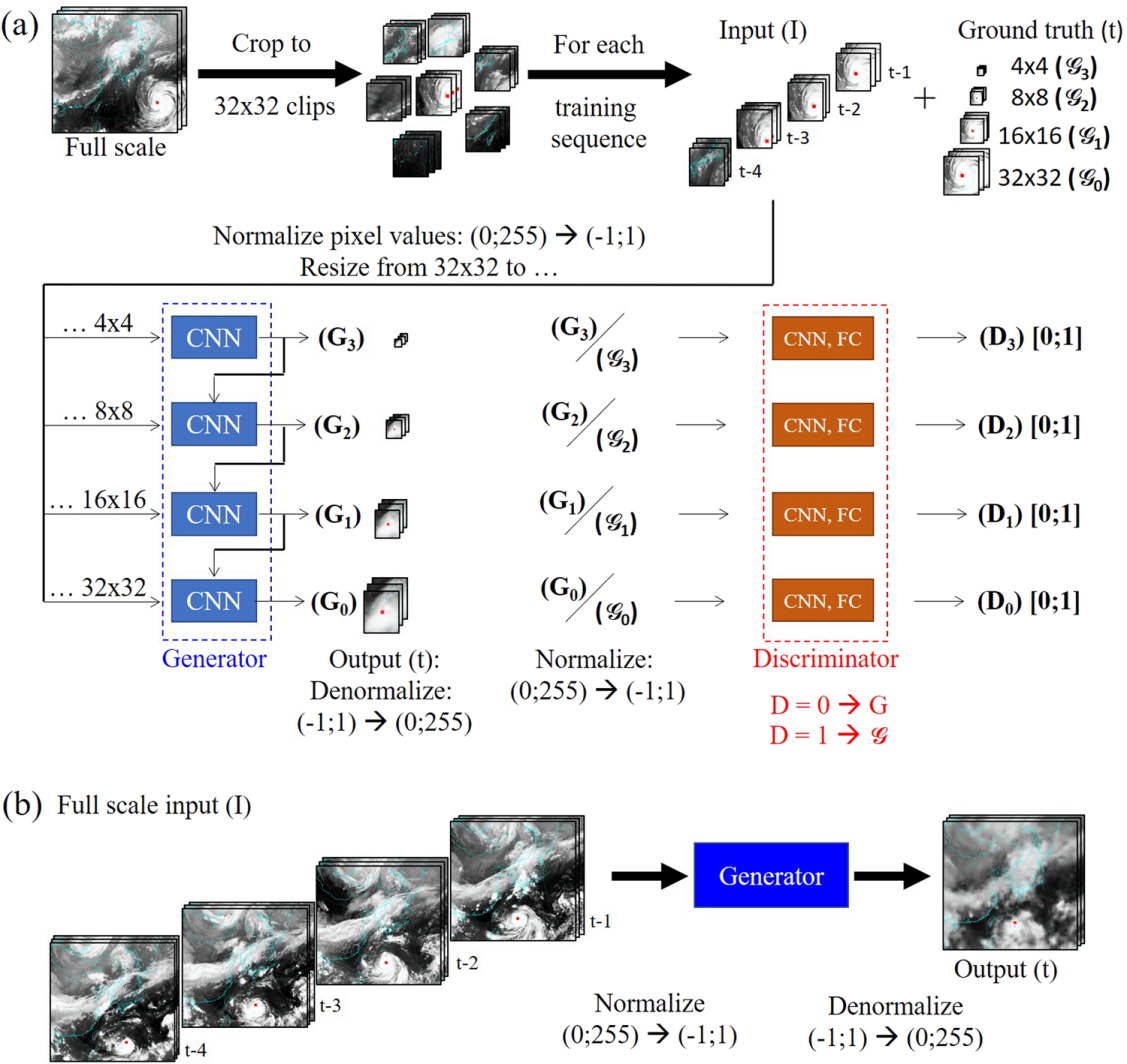
Overall structure of the utilized GAN for (**a**) training and (**b**) testing (Source of satellite images: Korean Meteorological Administration (KMA). http://www.weather.go.kr/weather/images).

The deep learning methodology is different for the training and the testing steps. Figure 12(a) shows the concept of adversarial training. For simplicity, a case is shown where only satellite images function as input. As mentioned previously in this work, each full scale satellite image consists of three color channels (RGB). They are represented throughout Fig. 12 by three overlapped layers. Before using the training data as input images to the GAN they get cropped to a total number of 5,000,000 clips with a pixel size of 32 × 32. One clip contains a set of *m* consecutively cropped satellite images from the past (input, *I*) and one ground truth image.

An important hyperparameter in this study is the number of input images (denoted by *m*). A high number of *m* increases memory usage and therefore the learning time. A low number might cause a shortage of input information for the GAN. Three cases with *m* = 2, 4, and 6 are trained and tested. Using currently available satellite images, the computation with *m* = 4 is found to produce the best result. In case of *m* = 2, temporal variation of a typhoon seems not to be sufficiently captured, while in case of *m* = 6, the result is deteriorated due to reduced numbers of training data sets. Considering, for example, typhoon Maemi with 16 sequences, using *m* = 4 allows to start training from sequence 5 and provides a number of 12 training sequences. The choice of *m* = 6, on the other hand, only allows 10 training sequences, starting from sequence 7.

Cropped training clips are trained using variations of deep learning networks in a GAN-based video modeling architecture^16^. This architecture contains a pure convolutional neural network (CNN), so called generator, and a network that combines convolutional layers with fully connected layers (FC), so called discriminator. The generator network takes a clip, normalizes its pixel values from (0; 255) to (−1; 1), predicts a 32 × 32 part of a satellite image at a future occasion (output, *G*_0_), and denormalizes the predicted pixels from (−1; 1) to (0; 255). GANs are multi-scale networks, therefore the generator network generates images on different scales (*G*_0_, *G*_1_, *G*_2_, *G*_3_). Generated images with pixel sizes of 4 × 4, 8 × 8, and 16 × 16 function as additional inputs for the next bigger scale, as pointed out in Fig. 12(a). This helps to keep long-range dependencies and preserves high resolution information throughout the convolutional learning. The discriminator network, on the other hand, tries to classify between the generated and the ground truth images of each scale. The output layer provides a binary output (*D*), where 0 stands for a generated image and 1 means the ground truth image. Testing steps are done with full scale test data, containing images with 250 × 238 pixels. They are not cropped. As visualized in Fig. 12(b), input images are taken and normalized by the generator network which generates a full scale image at a future occasion and denormalizes it.

The pixel size of 32 × 32 is selected for efficient usage of GPU memory. Even the network is trained with small size clips (32 × 32), it can eventually learn the same weights of the network that are learned with larger size clips. This is because kernel sizes for convolutions are under 7 × 7, so the network learns spatial characteristics under the space size of 7 × 7 pixels. However, during tests, typhoon satellite images on larger size (250 × 238) patches are predicted. This is possible because of the fully convolutional architecture of the generator.

The configuration of the deep learning network is summarized in Table 2 (see^16^ for detailed algorithms). An open source code^36^ is employed with modifications in input channels so that it is capable of changing the number of prior sequences and of accounting for additional variables such as velocity fields. The number of channels (*ch*) depends on the type of input data. If the GAN takes satellite images only as input data, three channels are needed. If images of zonal and meridional velocity fields are added to the input set, *ch* increases to 5. The network is trained from scratch.

**Table 2:** Configuration of the deep learning network.

Generator model (fully convolutional architecture)			
Convolution layers (top row: feature map sizes, bottom row: kernel sizes)			
**For generating** ***G***_**0**_	**For generating** ***G***_**1**_	**For generating** ***G***_**2**_	**For generating** ***G***_**3**_
ch × (m + 1), 128, 256, 512, 256, 128, 3	ch × (m + 1), 128, 256, 512, 256, 128	ch × (m + 1), 128, 256, 128, 3	ch × m, 128, 256, 128, 3
7 × 7, 5 × 5, 5 × 5, 5 × 5, 5 × 5, 7 × 7	5 × 5, 3 × 3, 3 × 3, 3 × 3, 3 × 3, 5 × 5	5 × 5, 3 × 3, 3 × 3, 5 × 5	3 × 3, 3 × 3, 3 × 3, 3 × 3
**Discriminator model (convolution layers** → **max pooling layers** → **fully connected layers)**			
**For output** ***D***_**0**_	**For output** ***D***_**1**_	**For output** ***D***_**2**_	**For output** ***D***_**3**_
ch, 128, 256, 512, 128	ch, 128, 256, 256	ch, 64, 128, 128	ch, 64
7 × 7, 7 × 7, 5 × 5, 5 × 5	5 × 5, 5 × 5, 5 × 5	3 × 3, 3 × 3, 3 × 3	3 × 3
**Max pooling layers (kernel sizes)**			
2 × 2	2 × 2	2 × 2	2 × 2
**Fully connected layers (neuron numbers)**			
1024, 512, 1	1024, 512, 1	1024, 512, 1	512, 256, 1

The number of channels (ch) depends on the type of input data. If the GAN takes satellite images only as input data, three channels are needed. If images of zonal and meridional velocity fields are added to the input set, ch increases to 5. The number of input images (*m*) defines the temporal range of information from which the GAN learns. For the results in Cases A and B, *m* is set to 4, which means the GAN learns from information of the past 24 hours (4 × 6 hours).

The generator model is trained to minimize a combination of loss functions as follows:

1 $${L}_{generator}=1/4\,{\sum }_{k=0}^{3}{\lambda }_{l2}{L}_{2}^{k}+{\lambda }_{gdl}{L}_{gdl}^{k}+{\lambda }_{adv}{L}_{adv}^{G,k},$$

where *λ*_*l*2_ = 1, *λ*_*gdl*_ = 1, and *λ*_*adv*_ = 0.05. Let *G*_*k*_(*I*) be the predicted image from a convolutional neural network of *G*_*k*_ and ${{\mathscr{G}}}_{k}(I)$ be a $\frac{1}{{2}^{k}}$ resized image from the provided ground truth image. The ${L}_{2}^{k}$ loss function evaluates the explicit difference between the predicted and provided images as

2 $${L}_{2}^{k}=||{G}_{k}(I)-{{\mathscr{G}}}_{k}(I)|{|}_{2}^{2}.$$

Generative techniques for video modeling are known to suffer from the blurriness^16,37,38^. To reduce the blurriness, Mathieu *et al*.^16^ proposed a gradient difference loss function (GDL) and reported that a GAN with the GDL improves the blurriness compared to other methods including a recurrent neural network based method proposed by Ranzato *et al*.^38^. The GDL, ${ {\mathcal L} }_{gdl}^{k}$, compares the difference between gradients of the predicted and provided images as

3 $$\begin{array}{c}{L}_{gdl}^{k}={\sum }_{i=0}^{{n}_{x}-2}{\sum }_{j=0}^{{n}_{y}-2}\{||{{\mathscr{G}}}_{k}{(I)}_{(i+1,j+1)}-{{\mathscr{G}}}_{k}{(I)}_{(i,j+1)}|-|{G}_{k}{(I)}_{(i+1,j+1)}-{G}_{k}{(I)}_{(i,j+1)}||\\ \,\,\,\,\,\,\,\,+||{{\mathscr{G}}}_{k}{(I)}_{(i+\mathrm{1,}j+\mathrm{1)}}-{{\mathscr{G}}}_{k}{(I)}_{(i+\mathrm{1,}j)}|-|{G}_{k}{(I)}_{(i+\mathrm{1,}j+\mathrm{1)}}-{G}_{k}{(I)}_{(i+\mathrm{1,}j)}||\},\end{array}$$

where *n*_*x*_ and *n*_*y*_ are numbers of pixel in the width and the height of an image, and (*i*, *j*) is a pixel coordinate. ${ {\mathcal L} }_{adv}^{G,k}$ loss function is applied to support the generator model to delude the discriminator network by generating images which are indistinguishable from the provided ground truth images as

4 $${L}_{adv}^{G,k}={L}_{bce}({D}_{k}({G}_{k}(I)),1),$$

where *L*_*bce*_ is the binary cross entropy loss function defined as

5 $${L}_{bce}(a,b)=-\,b\mathrm{log}(a)-\mathrm{(1}-b)\mathrm{log}\,\mathrm{(1}-a),$$

for scalars *a* and *b* between 0 and 1.

The discriminator model is trained to minimize a loss function as follows:

6 $${L}_{discriminator}=\frac{1}{4}\,{\sum }_{k=0}^{3}[{L}_{bce}({D}_{k}({{\mathscr{G}}}_{k}(I\mathrm{)),1)}+{L}_{bce}({D}_{k}({G}_{k}(I\mathrm{)),0)}]\mathrm{.}$$

This supports the network not to be deluded by the generator model by extracting important features of typhoons in an unsupervised manner.

For each testing, the generator model is fed with *m* consecutive full scale satellite images from the past and generates a full scale typhoon satellite image at future occasion (see Fig. 12(b)).

### Errors in the prediction of typhoon centers

In the current study two different errors are investigated. The first one describes the distance between the predicted coordinate (*ϕ*_*pred*_, *λ*_*pred*_) and the real coordinate (*ϕ*_*real*_, *λ*_*real*_), named as an absolute error (*E*). *E* is calculated in kilometers (km) by applying the haversine formula^39^, with the earth radius R taken at the location of the real coordinate:

7 $$E=2R\arcsin \sqrt{{\sin }^{2}\,(\frac{{\varphi }_{pred}-{\varphi }_{real}}{2})+\,\cos \,{\varphi }_{real}\,\cos \,{\varphi }_{pred}\,{\sin }^{2}\,(\frac{{\lambda }_{pred}-{\lambda }_{real}}{2})}.$$

However, the absolute error does not give sufficient information about the prediction quality. Tracks of slowly moving typhoons, for example, are more difficult to predict than tracks of fast ones. It is therefore necessary to introduce a relative error (*E*_*rel*_), that is calculated as follows:

8 $${E}_{rel}(t)=\frac{E}{2R\arcsin \sqrt{{\sin }^{2}(\frac{{\varphi }_{real}(t)-{\varphi }_{real}(t-6h)}{2})+\cos \,{\varphi }_{real}(t)\cos {\varphi }_{real}(t-6h){\sin }^{2}(\frac{{\lambda }_{real}(t)-{\lambda }_{real}(t-6h)}{2})}}.$$

This type of error considers the ratio between *E* and the distance that a typhoon has traveled over the last 6 hours, where *t* stands for the time of a certain sequence.

## Data Availability

All the data used in the present work are available upon request.
